# Knowledge and Perception of Falls among Community Dwelling Elderly: A Study from Southern Sri Lanka

**DOI:** 10.1155/2018/7653469

**Published:** 2018-05-29

**Authors:** Nirmala Gamage, Nirmala Rathnayake, Gayani Alwis

**Affiliations:** ^1^Department of Nursing, Faculty of Allied Health Sciences, University of Ruhuna, Galle, Sri Lanka; ^2^Department of Anatomy, Faculty of Medicine, University of Ruhuna, Galle, Sri Lanka

## Abstract

The knowledge and perception of falls facilitate a better pathway to improve the health status among the elderly. Knowledge and perception of falls among community dwelling elderly were assessed in 300 participants (175 females) aged 65 years and above using an interviewer-administered questionnaire. Mean (SD) age of the participants was 73.0 (6.7) years. Majority (72%) knew some biological factors, and 60% knew environmental and behavioral factors which increase the risk of falls. Among 300 participants, 18% had poor, 61% had average, and 21% had good knowledge on falls. The mean (SD) knowledge was 48.14 (19.13). The most frequent (49%) information source was television. Significant associations were found between age (*p* = 0.002) and educational status (*p* < 0.001) with level of knowledge regarding falls. Individuals, 25.4% with good knowledge, 32.2% with average knowledge, and 51.9% with poor knowledge, had experienced falls during the previous 12 months (*p* = 0.007). Regarding perception of falls, 20.3% (*n* = 61) had negative perception and 79.7% (*n* = 239) had positive perception. Significant associations were found between gender (*p* = 0.01), age (*p* = 0.04), and level of education (*p* < 0.001) with perception of falls. This study revealed that the community dwelling elders had average knowledge and positive perception regarding falls and preventive measures, emphasizing the importance of falls prevention awareness programs.

## 1. Introduction

Falls and fall-induced injuries in elderly people are major public health problems in most of the regions of the world. Fall is defined as “in-advertently coming to rest on the ground, floor or other lower level, excluding intentional change in position to rest in furniture, wall or other objects” [1]. Falls in older age may cause disabilities and morbidities; as a result, people suffer a lot with a huge burden to the family, health sector, and economy of the country. Many elderly people experience emotional problems such as loss of confidence, fear, and anxiety following falls which may lead to lack of motivation to do day-to-day normal activities in life [2].

Each year 28–35% of people over 65 years and 32–42% of people over 70 years experience falls [1]. A hospital-based study of elderly people in Colombo, Sri Lanka, reported that 23.3% of people over 65 years were subjected to falls within a year [3]. Risk factors for falls among elderly people are multifactorial, including complex interaction of biological, behavioral, environmental, and socioeconomic factors [1].

A study done by Laing and his group revealed that 62% of participants identified falls as a very urgent issue facing older adults, 69% had identified gait related safe activities, and only 21% were concerned about improvement of home safety in order to prevent falls [4]. In the same study, one-third (38%) of participants had good knowledge about recommended falls prevention practices, whereas 58% perceived themselves with average knowledge. However, when considering the perception towards falls, 34% of participants had considered falls as a least important problem [4].

According to the results of a self-administered survey conducted by American Physical Therapy Association, majority (86%) of participants considered falling as a major preventable health problem with safe environmental factors [5]. However, in an Indian study, with perspectives from different geographical locations, 88% of participants believed that older people do fall and there was no option to prevent falls [6]. Furthermore, elderly people had a belief that falls are an unavoidable consequence of aging and some people did not perceive their personal vulnerability for falls [7].

Another previous community based descriptive study carried out in Sri Lanka revealed that 48.6% had perceived falls among elderly as a significant health problem [8]. An Australian study revealed that older people preferred messages emphasizing the importance of good health and independence rather than falls prevention [9].

Even though falls among elderly people is a well-recognized topic in both medical and nursing literature under various headings, it has not been prioritized for research in many regions of the developing world [1]. Knowledge and perception of falls have positive influence on falls prevention or reduce the risk of falls among elderly people. Therefore, current study was aimed to evaluate the knowledge and perception of falls and related injuries among elderly people in a rural community in Southern Sri Lanka aiming to reduce risk of falls and fall-induced injuries among elderly people.

## 2. Material and Methods

### 2.1. Study Design and Sampling

This cross-sectional household survey was conducted, during April and May 2015, with the participation of 300 community dwelling individuals aged 65 years and above from Nagoda Divisional Secretariat (DS) Area, Galle, Sri Lanka. The cluster sampling method was used to achieve the required study sample from five out of 53 Grama Niladhari (GN) divisions that are under the administration of Nagoda DS office covering 62176 inhabitants with 9024 (14.51%) elderly people aged 65 years and above. Sixty participants were taken as the cluster size as households within each GN division were randomly selected based on the assumption that at least one ambulatory person aged 65 years or older lives in each household. Individuals without any acute illness and who can understand the questionnaire were considered as the inclusion criteria.

### 2.2. Data Collection

The data collection was conducted at the recruited participants' residence from the individuals who met the inclusion criteria with the support of a family member for the accuracy of data, especially in older elderly people with hearing and/or memory impairment. A convenient and adequate time was given for responding to the questions, which did not interfere with their comfort and day-to-day activities. Pretested, interviewer-administered questionnaire was applied by a single investigator to assess sociodemographic profile and different significant aspects related to the knowledge and perception of falls. Sociodemographic profile included age, gender, marital status, educational status, previous employment status, and living companion. Socioeconomic factors such as income and accessibility of health and social care services were also obtained. Individuals were asked whether they had experienced at least one fall during the previous 12-month period.

Knowledge about falls was assessed on several aspects. Knowledge on WHO defined four major risk factors for falls such as biological, environmental, behavioral, and socioeconomic status [1], and disease conditions that increase the risk of falling, consequences of falls, and preventive measures of falls were evaluated under 19 statements. The sources of information about falls and preventive measures were also obtained. Perception of falls was assessed under eight statements in a five-point Likert scale, ranging from strongly agree to strongly disagree. There were three negatively and five positively expressed statements. These statements were developed by the investigators using the statements which had been used to assess perception regarding falls among elderly people in previous studies [4, 6].

### 2.3. Statistical Analysis

Statistical analysis was done using Statistical Package of Social Sciences (SPSS) version 20.0. Descriptive statistics were performed including mean and Standard Deviation (SD). Pearson's chi-square test was used to assess the association between categorical variables. Statistical significance was set at *p* < 0.05. Each question was numerically coded for statements on knowledge and perception of falls to obtain the knowledge and perception scores.

*Knowledge Score*. Maximum mark for a question was 5. The total maximum marks which could be obtained were 95 with a minimum of zero. The total score was categorized into three groups. Individuals who scored less than one-third of points (0–31) were considered as having poor knowledge, those who scored 32–63 points were considered as having average knowledge, and those who scored 64–95 points were considered as having good knowledge.

*Perception Score*. Each statement was rated based on the Likert scale ranging from 1 to 5. The total maximum marks which could be obtained were 40 with a minimum of 08. Individuals who scored 08–24 were considered as having negative perception and those who scored 25–40 points were considered as having positive perception.

### 2.4. Ethical Considerations

The ethical approval for this study was obtained from the Ethical Review Committee, Faculty of Medicine, University of Ruhuna, Sri Lanka. Written informed consent was obtained from each study participant before the administration of questionnaire.

## 3. Results

### 3.1. Sociodemographic Profile

The sociodemographic characteristics of the study participants are shown in Table 1. The sample consisted of 300 participants aged 65 years and over (age range; 65–99 years) with a mean (SD) age of 73.0 (6.7) years. 58% of participants were females and 42% were males. Majority (89.3%) lives with their spouse or children and only 10.7% live alone. Majority (79.3%) was not currently employed and the women were mainly involved in household work. Majority (61.3%) had education beyond the secondary level, whereas 31% of study participants had only primary education and 7% had no schooling. Among the participants, 34.3% (*n* = 103) reported that they experienced at least one fall during the previous 12-month period and 35.9% reported related injuries (Table 1).

### 3.2. Knowledge Regarding Falls

**Figure 1:**
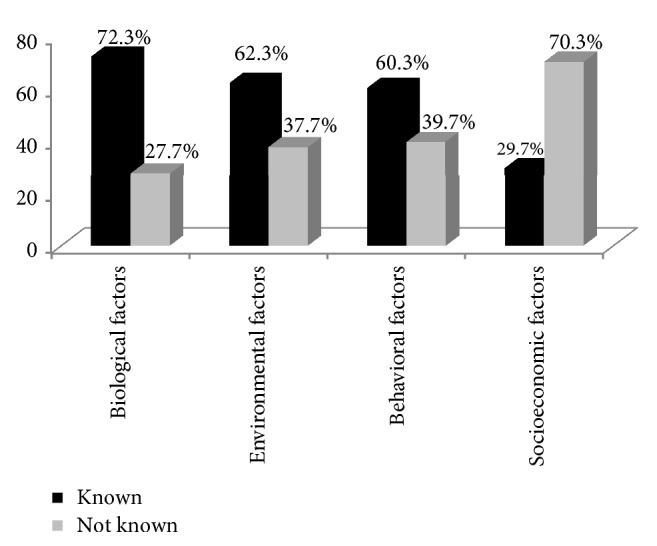
Knowledge on major risk factors for falls (*n* = 300).

Knowledge regarding different aspects of falls is shown in Table 2 and Figure 1. Out of 300 participants, half (50.7%) knew that falls and related injuries are the leading causes of hospital admission among elderly people, whereas 49.3% considered it as a least contributing cause for elderly hospitalization. Although, majority (72.7%) considered proper nutrition as a protective factor for falls, only 55.7% were aware of the importance of active lifestyle to reduce the risk of falls among elderly people. Majority (73.7%) recognized the protective environmental factors against falls such as good lighting and home safety and 56% of participants knew that following proper medical advice helps to minimize the chances of falls due to side effects of medications. Majority (82.3%) knew that hypertension increases the risk for falls; however, only 31.3% believed that diabetes mellitus increases the risk for falls. Regarding the awareness on consequences of falls, majority (77%) believed that falls lead to reduced physical mobility and nearly half (47%) considered restriction of day-to-day activities. However, only 17.3% believed the possibility of social isolation due to disabilities following falls (Table 2).

Around 72% of participants were able to name at least one or two biological factors such as age and chronic medical conditions which increase the risk of falls. Around 60% knew that environmental and behavioral factors contribute to falls, but only 29.7% knew the contribution of poor socioeconomic factors in increasing the risk of falls (Figure 1).

Considering overall level of knowledge of the 300 participants, 18% (*n* = 54) of individuals had poor knowledge, 61% (*n* = 183) had average knowledge, and 21% (*n* = 63) had good knowledge on falls, related injuries, and preventive measures. The mean (SD) knowledge of the participants was 48.14 (19.13).

There were statistically significant associations between knowledge on falls with age (*p* = 0.002), educational status (*p* < 0.001) (Table 3), and history of falls during previous 12-month period (*p* = 0.007) (Table 4). Further, 25.4% of individuals with overall good knowledge, 32.2% of individuals with average knowledge, and 51.9% with poor knowledge regarding falls reported falls during previous 12-month period (Table 4).

**Figure 2:**
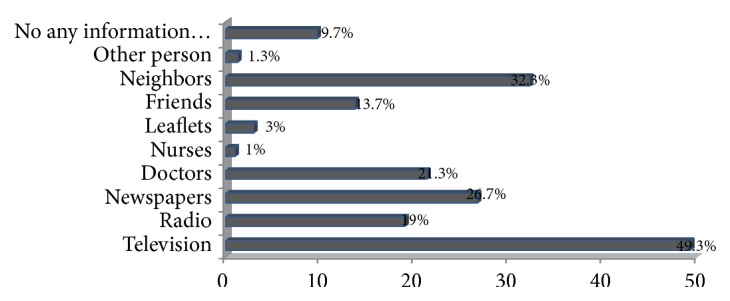
Sources of information regarding falls knowledge.

Nearly half (49.3%) of the participants obtained information on falls and preventive measures via television and 32.3% of participants shared knowledge on falls and related injuries with their neighbors' experiences. 26.7% had read newspaper articles regarding falls. Only 1% of participants had gained knowledge from nurses and 9.7% were unable to gain information from any source (Figure 2).

### 3.3. Perception of Falls

Perceptions on falls are shown in Table 5. Of the 300 study participants, 37.3% perceived that older people fall and there's nothing to be done in preventing those falls, but 11.7% strongly disagreed with that statement. 22.7% of individuals did not believe that they were having a risk for falls. Although 33.3% of participants agreed that they did not worry about falls, 29.3% disagreed and 17.3% strongly disagreed with that statement. While half (49.7%) of individuals strongly perceived that the safety of their house was good, only 6.4% of individuals disagreed or strongly disagreed with that statement. Although 15.3% of individuals strongly agreed and 37.3% agreed with the statement that they need to do fall prevention activities, 33% disagreed or strongly disagreed with the statement and believed that they were not weak. Individuals (21%) strongly believed that falls prevention interventions given after the first fall may be helpful to prevent recurrent falls. Majority (62%) strongly agreed on the importance of falls prevention knowledge training programmes and only 1% (*n* = 3) of participants disagreed with the statement. Majority (82.7%) strongly perceived that paying attention to correct medical conditions as a considerable matter, only a single individual had neutral perception and no one disagreed on that statement (Table 5).

Of the 300 participants, 20.3% (*n* = 61) had negative perception and 79.7% (*n* = 239) had positive perception regarding falls. There were statistically significant associations between perception of falls with gender (*p* = 0.01), age (*p* = 0.04), and level of education (*p* < 0.001) (Table 6). But there was no statistically significant association between perceptions of falls with history of falls (Table 7).

## 4. Discussion 

The current study evaluated the knowledge and perception of falls, related injuries, and preventive measures among elderly people in a rural community in Sri Lanka and revealed that the majority of study participants had an average level of knowledge and a positive perception of falls.

Nearly half of the participants in the study sample knew that falls are the leading cause of hospital admissions among elderly people. This is consistent with the findings of the study conducted in the Colombo District, which revealed that 48.6% participants had considered falls among elderly people as a significant health problem [8]. These findings reflect the need to encourage elderly people in both urban and rural areas in Sri Lanka to consider falls as an important health concern and to reduce the economic burden of the country that may arise due to falls.

A majority of them considered that proper nutrition, active lifestyle, and protective environmental factors had an effect on falls. Yet, although the majority of subjects were aware about biological, behavioral, and environmental factors that contribute to increased risk of falls, there was a lack of knowledge regarding socioeconomic factors. This may be due to indirect contribution of socioeconomic factors towards falls.

When considering the biological factors, even though most of the elderly people had favorable knowledge about hypertension increasing the chance of falls, a majority did not believe that diabetes mellitus contributes to increase the risk of falls. The reason may be due to lack of awareness regarding symptoms and complications of such chronic disease conditions.

Young elderly people (65–74 years) and individuals, who were educated beyond primary education, had an overall good level of knowledge regarding falls and related injuries. Significant associations were found between, age, and educational status with the level of knowledge. Educated people having a tendency to pay more attention to gain awareness on health issues and young elderly individuals having more social interaction than older elderly people could be the plausible explanations for these observations.

The findings of the majority of participants with average knowledge on falls and related injuries in our study are consistent with a cross-sectional survey done in the USA to assess the fall prevention knowledge, attitude, and practices of community stakeholders and older adults [4]. This study revealed that 58% of individuals were fairly knowledgeable about falls and preventive practices [4]. However, it contradicted with the findings of another cross-sectional study conducted in South India, which revealed poor knowledge of Indian elderly people regarding falls and preventive measures [6].

In Sri Lanka, the most frequent information sources on falls and preventive measures were found to be television and neighbors' experiences. Least frequent was awareness from nurses. This reflects the inadequate level of nurse-client relationship and deficit of community health nursing services in the country. Further, it emphasizes “health education” as a vital responsibility which needs to be developed in the health care system in Sri Lanka. A significant proportion (9.7%) of elderly people was not being able to expose any kind of information source which could be due to isolation or lack of access to resources.

With regard to the perception of falls, 39.7% disagreed or strongly disagreed with the statement “older people do fall and nothing can be done to prevent falls.” However, the previously mentioned Indian study reported that only 12% of participants disagreed with the same statement [6]. Sri Lankan elderly people have shown a positive attitude than Indians on this specific health matter, which may be due to sociocultural and educational differences between the two countries.

In our study, the majority of elderly individuals agreed that “the interventions given after the first fall can prevent recurrent falls” which is consistent with the previous study reported from India [6]. Further, a majority strongly agreeing with the statement “paying attention to correct my medical condition is very important” in our study may be due to improved health awareness and free health services in Sri Lanka.

We observed that a majority of study participants had positive perception towards falls and significant associations were found between gender and educational status. Majority of elderly females and individuals educated beyond primary education had positive perception of falls and the reasons for these observations are questionable.

In our study, nearly half of the individuals with poor knowledge had reported history of falls and in contrast only a small proportion of individuals with good knowledge had reported previous fall event. Further, there was a statistically significant association between knowledge and history of falls during the previous 12-month period. This suggests that having a good knowledge results in lesser chances of falling and related injuries. Even though there was no significant association, most of the individuals with positive perception did not report history of falls during the previous 12-month period. It indicates that the individuals with positive perception always pay attention to personal susceptibility and follow preventive measures to get rid of falls and related consequences.

## 5. Conclusions

In this cross-sectional study, community dwelling older people had average level of knowledge and positive perception towards falls and related injury prevention. The most frequent awareness information source on falls was television. Significant associations were found between age, educational status, and history of falls with the level of knowledge. Gender, age, and educational status were significantly associated with the perception. This study emphasizes the importance of increasing the level of knowledge on falls, related injuries, and preventive measures.

## Figures and Tables

**Table 1:** Sociodemographic profile of the study sample (*n* = 300).

Socio demographic characteristics	Female *n* (%) (*n* = 175)	Male *n* (%) (*n* = 125)	Total *n* (%) (*n* = 300)
*Age group*			
65–74 y	122 (69.7)	78 (62.4)	200 (66.7)
75 y and above	53 (30.3)	47 (37.6)	100 (33.3)

*Level of education*			
No schooling	10 (5.7)	13 (10.4)	23 (7.7)
Primary education	60 (34.2)	33 (26.4)	93 (31.0)
Secondary education	81 (46.3)	67 (53.6)	148 (49.3)
Upper secondary or above	24 (13.7)	12 (9.6)	36 (12.0)

*Marital status*			
Married	98 (56.0)	103 (82.4)	201 (67.0)
Single	14 (8.0)	05 (4.0)	19 (6.3)
Widowed	57 (32.6)	16 (12.8)	73 (24.3)
Divorced	03 (1.7)	01 (0.8)	04 (1.3)
Separated	03 (1.7)	00 (0.0)	03 (1.0)

*Living companion*			
With spouse or children	151 (86.3)	117 (93.6)	268 (89.3)
Alone	24 (13.7)	08 (6.4)	32 (10.7)

*Previous employment status*			
Employed	27 (15.4)	35 (28.0)	62 (20.7)
Not employed	148 (84.6)	90 (72.0)	238 (79.3)

*Monthly income (LKR)*			
<10000	73 (41.7)	59 (47.2)	132 (44.0)
10000–20000	94 (53.7)	55 (44.0)	149 (49.7)
20000–50000	8 (4.6)	11 (8.8)	19 (6.3)

*Satisfy about accessibility to health and social care services*			
Yes	84 (48.0)	67 (53.6)	151 (50.3)
No	91 (52.0)	58 (46.4)	149 (49.7)

*Experienced at least one fall during previous 12 months period*			
Yes	71 (40.6)	32 (25.6)	103 (34.3)
No	104 (59.4)	93 (74.4)	197 (65.7)

*Experienced fall related injuries *(*n* = 103)			
Yes	27 (38.0)	10 (31.2)	37 (35.9)
No	44 (62.0)	22 (68.8)	66 (64.1)

1 LKR = 150 USD			

**Table 2:** Statements that evaluate the knowledge on falls (*n* = 300).

Statement	Known	Not known
	*n* (%)	*n* (%)
Falls and related injuries are the leading cause of hospital admission among elderly people.	152 (50.7)	148 (49.3)

Proper nutrition is very important to protect from falls.	218 (72.7)	82 (27.3)

Exercise and active lifestyle reduce the chances of falls.	167 (55.7)	133 (44.3)

Following medical advice helps to minimize the chances of falls due to side effects of drugs.	168 (56.0)	132 (44.0)

Good lighting reduces the risk of falls.	221 (73.7)	79 (26.3)

Aware of at least two commonest sites for fall related fractures.	251 (83.7)	49 (16.3)

Aware of at least two food items which are helpful for healthy bones.	183 (61.0)	117 (39.0)

Aware of medical conditions which increase the risk for falls.		
Hypertension	247 (82.3)	53 (17.7)
Diabetes mellitus	94 (31.3)	206 (68.7)
Arthritis	77 (25.7)	223 (74.3)
Ischemic Heart Disease	40 (13.3)	260 (86.7)

Aware of the consequences of falls.		
Reduced mobility	231 (77.0)	69 (23.0)
Restriction of day today activities	141 (47.0)	159 (53.0)
Social isolation	52 (17.3)	248 (82.7)
Depression	63 (21.0)	237 (79.0)

**Table 3:** Association of knowledge of falls with demographic characteristics (*n* = 300).

Demographic characteristic	Knowledge on falls			*p* value
	Poor	Average	Good	
	*n* (%)	*n* (%)	*n* (%)	
Gender				
Female	31 (17.7)	105 (60.0)	39 (22.3)	0.81
Male	23 (18.4)	78 (62.4)	24 (19.2)	
Age				
65–74 y	26 (13.0)	125 (62.5)	49 (24.5)	0.002
75 y and above	28 (28.0)	58 (58.0)	14 (14.0)	
Level of education				
Primary education or less	42 (36.2)	68 (58.6)	6 (5.2)	<0.001
Beyond primary education	12 (6.5)	115 (62.5)	57 (31.0)	

**Table 4:** Association of level of knowledge with history of falls (*n* = 300).

Level of knowledge	History of falls during previous 12 months		*p* value
	Yes (*n* = 103)	No (*n* = 197)	
	*n* (%)	*n* (%)	
Poor	28 (51.9)	26 (48.1)	0.007
Average	59 (32.2)	124 (67.8)	
Good	16 (25.4)	47 (74.6)	

**Table 5:** Statements that describe the perception towards falls (*n* = 300).

Statement	Strongly agree	Agree	No idea	Disagree	Strongly disagree
	*n* (%)	*n* (%)	*n* (%)	*n* (%)	*n* (%)
Older people fall and there is nothing that can be done to prevent falls.	112 (37.3)	51 (17.0)	18 (6.0)	84 (28.0)	35 (11.7)

It is not possible for me to fall down and get injured or fractured.	68 (22.7)	96 (32.0)	48 (16.0)	64 (21.3)	24 (8.0)

I do not worry about falling down and getting injured.	36 (12.0)	100(33.3)	24 (8.0)	88 (29.3)	52 (17.3)

The safety of my house is very good for preventing falls.	149 (49.7)	81 (27.0)	51 (17.0)	17 (5.7)	2 (0.7)

I am weak and need to follow fall prevention activities.	46 (15.3)	112 (37.3)	43 (14.3)	69 (23.0)	30 (10.0)

The intervention given after first fall can prevent recurrent falls.	63 (21.0)	119 (39.7)	100 (33.3)	18 (6.0)	0 (0.0)

Carrying out knowledge training programmes on fall induced injury in the community is a great necessity.	186 (62.0)	86 (28.7)	25 (8.3)	3 (1.0)	0 (0.0)

Paying attention to correct my medical conditions is very important to get rid of falls.	248 (82.7)	51 (17.0)	1 (0.3)	0 (0.0)	0 (0.0)

**Table 6:** Association of perception of falls with demographic variables (*n* = 300).

Demographic characteristic	Perception regarding falls		*p* value
	Negative	Positive	
	*n* (%)	*n* (%)	
Gender			
Female	27 (15.4)	148 (84.6)	0.01
Male	34 (27.2)	91 (72.8)	
Age			
65–74 y	34 (17.0)	166 (83.0)	0.04
75 y and above	27 (27.0)	73 (73.0)	
Level of education			
Primary education or less	42 (36.2)	74 (63.8)	<0.001
Beyond primary education	19 (10.3)	165 (89.7)	

**Table 7:** Association of perception with the history of falls (*n* = 300).

Level of perception	History of falls during previous 12 months		*p* value
	Yes (*n* = 103)	No (*n* = 197)	
	*n* (%)	*n* (%)	
Negative	26 (42.6)	35 (57.4)	0.12
Positive	77 (32.2)	162 (67.8)	

## Data Availability

The data used to support the findings of this study are available from the corresponding author upon request.
